# Accordance of Registered Drug Packages with Guideline-Recommended Treatment Durations for Community-Acquired Pneumonia—A New Antibiotic Stewardship Target?

**DOI:** 10.3390/antibiotics13060546

**Published:** 2024-06-12

**Authors:** Martina Prusac, Maja Ortner Hadziabdic, Doris Rusic, Darko Modun

**Affiliations:** 1 Community Pharmacy Drazenovic, Ante Starcevica 9, 20350 Metkovic, Croatia; martinas4@hotmail.com; 2 Center for Applied Pharmacy, Faculty of Pharmacy and Biochemistry, University of Zagreb, Ante Kovacica 1, 10000 Zagreb, Croatia; mortner@pharma.hr; 3 Department of Pharmacy, University of Split School of Medicine, Soltanska 2A, 21000 Split, Croatia; drusic@mefst.hr

**Keywords:** community-acquired pneumonia (CAP), treatment guidelines, drug package

## Abstract

In most countries, antibiotics for oral administration are put on the market in fixed packages. When there is no exact unit dispensing of antimicrobials, drug pack size may influence prescribers’ choice of treatment duration. The aim of this study was to investigate the accordance of approved antibiotic packages with national guidelines for the treatment of community-acquired pneumonia (CAP). For the purpose of this study, criteria were developed to determine the accordance of approved antibiotic packages for treating CAP (criteria), which are based on recommendations from national guidelines for treating CAP. Subsequently, the accordance of approved antibiotic packages with the number of antibiotic doses resulting from the specified criteria was determined. Of 39 identified therapeutic option-package size combinations, 11 were found to be matched (28.2%), meaning there were no leftover medication units after completing therapy, and 28 were mismatched combinations (71.8%), indicating that there were excess doses of antibiotics remaining at the end of therapy. The results of this research showed a significant non-accordance of the approved antibiotic packages with the national guidelines for the treatment of CAP and, consequently, the creation of a large amount of residues of unit doses of antibiotics in the community.

## 1. Introduction

Antimicrobial resistance is already a serious social and economic problem [1]. The direct consequences of infection caused by resistant microorganisms can be serious and include longer disease duration, increased mortality, prolonged hospital stay, loss of protection for patients undergoing surgery and other medical procedures, and increased costs [2]. Globally, resistant infections are estimated to cause 700,000 deaths annually. It is estimated that failure to take the necessary steps could lead to millions of deaths worldwide and that antimicrobial resistance could cause more deaths than cancer by 2050. The World Bank warns that by 2050, infections caused by resistant microorganisms could cause economic damage comparable to the financial crisis of 2008 [1].

Antimicrobial resistance represents a global crisis that must be urgently addressed [2] because it could be the biggest challenge that the healthcare system faces in the 21st century [3]. If aligned global action does not begin immediately, the world could face a return to the pre-antibiotic era as common infections could once again become deadly [1,2].

Antimicrobial resistance is a complex problem shaped by numerous interrelated factors, especially the excessive and incorrect use of antimicrobial drugs [3]. Healthcare professionals play a vital role in preserving the effectiveness of antimicrobial drugs [2]. Mekker et al. report that despite published clinical guidelines and decades of efforts to change prescribing habits, antibiotics continue to be overused. Interventions such as prescriber and patient education, computerized clinical decision support, and financial stimulus have historically produced modest results in reducing antimicrobial resistance for acute respiratory infections [4]. Great hopes are placed on the concept of antimicrobial stewardship, which significantly contributes to the rational use of antimicrobial drugs with the aim of reducing antimicrobial resistance [5].

Respiratory infections of the lower respiratory system remain the deadliest infectious diseases, and in total diseases, they rank fourth according to the World Health Organization’s 2020 report on the 10 most common causes of death [6]. Pneumonia is common, and in older people, it is often a serious and fatal disease. Community-acquired pneumonia (CAP) is defined as pneumonia that is acquired outside the hospital. Most patients are treated on an outpatient basis, and approximately one third require hospitalization [7]. Despite powerful antibiotics and the use of additional and supportive therapy, pneumonias represent a therapeutic challenge even in the 21st century, and an important reason for this is the increasing occurrence of resistant microbiological agents [8].

Economic implications of antimicrobial misuse are not to be understated, especially considering the high economic burden of CAP, growing incidence with age, and ageing population [9]. Adherence to clinical guidelines for antibiotic treatment selection is associated with improved patient outcomes and sometimes a reduction in cost [10]. Studies evaluating antibiotics prescribed at hospital discharge have identified antibiotic overuse in CAP as common, reaching 70%, with an excessive duration of treatment occurring most frequently. This can result in longer hospital stays and more emergency department visits within 30 days of discharge, further driving the health-related costs for these patients [11].

An intervention with a number of antimicrobial stewardship measures that included interactive teaching and a prescribing app was undertaken in a hospital in Ireland after the management of CAP was identified as rather poor. In this study, compliance with guidelines has seen three-fold increase, and a shorter treatment duration was observed with the implementation of a number of measures for hospitalized patients with CAP [12]. In another study, physician-driven stewardship intervention was introduced to optimize treatment in hospitalized patients with CAP, and it reduced excess antibiotic days beyond the Infectious Diseases Society of America and American Thoracic Society recommended minimum duration from 3 to 0 days during intervention [13]. Furthermore, Italy is burdened with one of the top prescribing rates of antimicrobials in Europe. As an antimicrobial stewardship program measure, an implementation of a clinical pathway was proposed. This was a one-page decision support algorithm that summarized guidelines on the initiation of antibiotics, drug choice, and treatment duration in a pediatric emergency department. This intervention resulted in the decreased prescribing of broad-spectrum antibiotics and shorter recommended treatment duration for patients with a descriptive diagnosis of CAP [14]. 

Overall, research indicates that the greater the number of stewardship measures implemented, the lower antibiotic overuse is [15]. Enhancing electronic medicine prescribing with antimicrobial stewardship features can reduce days of antibiotic treatment [16].

Guidelines for the treatment of infectious diseases recommend evidence-based therapeutic options. Recommendations usually include the first and alternative choice of active substance, dose, dosing regimen, and duration of therapy. Recent evidence suggests that an extended duration of therapy may be unnecessary since many clinical studies have shown that shorter treatment regimens are equally effective. Despite the evidence, guidelines still recommend a relatively long duration of treatment, or they are unclear [17]. If the duration of therapy is not clearly recommended by the guidelines and the prescriber has to make a decision by themselves, they can be guided by the size of the package available on the market and, with that in mind, decide how long the therapy will last [18].

In most countries, antibiotics for oral administration are put on the market in fixed packages. When there is no exact unit dispensing of antimicrobials, drug pack size may influence prescribers’ choice of treatment duration. Moreover, authors suggest that studies analyzing and investigating factors influencing the behavior of prescribers are warranted [19]. Several authors suggest that assuring that the registered drug packs of antimicrobials are in accordance with treatment guidelines can add to other stewardship measures and improve adherence to treatment guidelines as the size of a registered antibiotic pack can indicate a default duration of treatment for prescribers [18,20,21]. 

The aim of this study was to investigate the accordance of approved antibiotic packages in the Republic of Croatia with national guidelines for the treatment of community-acquired pneumonia.

## 2. Results

The criteria established for the purposes of this study ultimately identified a total of 21 therapeutic options, as shown in Supplementary Table S1. Among these, cefuroxime axetil, cefpodoxime, moxifloxacin, azithromycin, and doxycycline had one therapeutic option each. Amoxicillin with clavulanic acid had two therapeutic options that differed in terms of the duration of therapy. Amoxicillin and levofloxacin had four therapeutic options each, which varied in strength and duration of therapy, while clarithromycin had six therapeutic options that differed in pharmaceutical form, strength, and duration of therapy.

For each active substance recommended in the guidelines for treating CAP, the available packages were determined and are shown in Table 1 according to their pharmaceutical form, strength, and number of doses per package.

The list of approved package sizes of antibiotics for oral use in Croatia, compiled for the purposes of this study, contains a total of 22 possibilities. Among these, doxycycline and levofloxacin each have one possibility. Amoxicillin has two possibilities that differ in strength. Amoxicillin with clavulanic acid and moxifloxacin also have two possibilities each, differing in package size. Azithromycin has two possibilities that differ in strength and package size. Cefuroxime axetil and cefpodoxime each have four possibilities that differ in strength and package size, while clarithromycin also has four possibilities that differ not only in strength and package size but also in pharmaceutical form.

This study identified a total of 39 combinations of therapeutic options outlined by the criteria and package sizes from the compiled list, as shown in Table 2. Of these, 11 were found to be matched (28.2%), meaning that there were no leftover medication units after completing therapy, and 28 were mismatched combinations (71.8%), indicating that there were excess doses of antibiotics remaining at the end of therapy. For amoxicillin, six combinations were identified, all of which were mismatched. For cefuroxime axetil and amoxicillin with clavulanic acid, four combinations each were identified, with one being matched and three being mismatched. One combination was identified for doxycycline, while four combinations were identified for cefpodoxime and levofloxacin, all of which were mismatched. Two combinations were identified for moxifloxacin, one of which was matched and the other mismatched. Two combinations were identified for azithromycin, both of which were matched. For clarithromycin, 12 combinations were identified, with six being matched and the remaining six being mismatched. The summary of the accordance of drug packs with the clinical guidelines is shown in Table 3.

## 3. Discussion

This study identified a total of 39 combinations of therapeutic options and attributed package sizes, of which 11 were found to be matched (28.2%) with no leftover medication units after completing therapy, and 28 were mismatched combinations (71.8%), indicating that there were excess doses of antibiotics remaining at the end of completing the treatment. All the identified combinations of the therapeutic options for amoxicillin, doxycycline, cefpodoxime, and levofloxacin were mismatched, and only the ones identified for azithromycin were completely matched. The results of this research are consistent with previous research on this topic. A recently published study investigated the accordance of registered oral drug packages in the indication of prostatitis with the published guidelines across different countries. In Croatia, a number of mismatches occurred; however, macrolides remained matched [20]. In a similar study conducted in the indication of *H. pylori*, the author speculated that poor matching in Croatia may be attributed to a smaller variety in registered drug package sizes in smaller markets [18]. 

Antibiotics are registered for different indications, so there is a possibility that the registered packages would be in accordance with other indications than CAP. This was confirmed by a study in which the authors aimed to pair registered drug packages with the published guidelines, but they found only great mismatching since they focused on only a couple of indications [21]. This was likely guided with the fact that most antibiotics prescribed in primary healthcare are prescribed for respiratory and urinary infections [22]. Moreover, CAP is referred to as one of the leading causes of morbidity and mortality of older adults [23]. Hence, the focus is set on improving treatment outcomes in CAP. There has been a debate about rationalizing the use of antibiotics in CAP as it is among the leading indications for antimicrobial prescribing. More often than not, the causative agent of CAP fails to be recognized, so this tends to result in antibiotic overtreatment [19]. A study estimating the appropriateness of antibiotics across hospitals in the United States reported that 79.5% of treatments for CAP are unsupported by medical records, with excessive treatment duration being the most frequent reason for such a classification [24]. A study conducted in the United Kingdom found that potential to reduce antibiotic use by 32% in patients with CAP [25]. Inappropriate prescribing for CAP was also reported in a Canadian study conducted in a community hospital’s emergency department [26].

Previous studies have found that there is low accordance of prescribed antibiotics with the guidelines. A Swiss study reported adherence to guidelines in CAP of 54% and prolonged duration of antibiotic treatment, with 44% of patients with CAP being discharged with oral antibiotics [27]. The inappropriate prescribing of antibiotics upon hospital discharge is well documented, with most patients receiving antibiotics longer than needed or recommended [28]. Studies evaluating antimicrobial days of treatment on hospital discharge have often found them to be excessive and most pronounced for CAP [29,30]. 

The mismatch between guideline-recommended duration of therapy and antibiotic pack size can mean that a significant amount of antibiotics are dispensed rather than administered for acute infectious diseases, resulting in antibiotic residues in the community. McGuire et al. conclude that there could be a large amount of antibiotics in excess in the community, considering that research determines the non-accordance of guidelines and package sizes much more often than the accordance of them [31], which was confirmed by the results of this research. Mukherjee et al. found comparable results and suggest that specialists, industry, and health policymakers consider antibiotic package sizes to devise steps to reduce residual antibiotics after antimicrobial treatment is completed [17]. 

Any deviation of the package size from the recommended duration of therapy by the guidelines and ultimately by the prescriber can lead to residual unit doses. Previous research indicates that antibiotic residues can become pharmaceutical waste or encourage the patient to use them on another occasion, thus contributing to improper use. If they become waste or are improperly applied, they contribute to the development of antimicrobial resistance [31,32,33].

Antibiotic residues were reported by 19% of UK households surveyed, according to a survey of 6,983 households. Prescriptions prescribed for packs for therapy longer than 6 days accounted for 61% of residues, and prescriptions prescribed for packs for therapy shorter than 3 days accounted for 6% of residues [34]. Given that the United Kingdom has a system of dispensing antibiotic therapy units, the excess of drug units was due to non-adherence. It is to be expected that the residues of antibiotic therapy are even greater in countries where fixed packages of antibiotics are issued, as is the case in the Republic of Croatia.

Machowska et al. emphasize the importance of preventing the creation of excess drugs in order to consequently prevent the previously mentioned problems. Some of the suggested strategies are educating patients about programs for returning excess drugs to community pharmacies, which is also recommended by the WHO, then harmonizing the size of the package with the recommended duration of therapy, and unit dispensing of antimicrobial therapy [32].

Accurately matching the registered pack size and duration of therapy according to the guidelines appears to be a rather ambitious strategy. Each antibiotic has been approved for different indications and a different duration of therapy for the same indication. In addition, guideline recommendations change relatively frequently in light of new clinical evidence. The implementation of this strategy would place a large financial burden on the pharmaceutical industry, and it is questionable how effective it would be, given that patient adherence is an important factor in generating excess drugs [31].

Some authors suggest unit dispensing as a strategy to prevent antibiotic residues and the consequent problems [35]. A recent international survey found that subjects living in states where fixed-pack antibiotics are dispensed are more likely to have an excess of antibiotics and continue to use them unsupervised, compared to subjects living in states where unit antimicrobial therapy is dispensed [36]. Previous research has shown that dispensing the exact number of unit doses of antibiotics reduces costs for insurers, reduces the amount of unused antibiotics in excess, and has a positive effect on the environment and adherence to therapy [35].

However, it remains unclear whether the implementation of unit dispensing is still possible given the recent efforts of the EU in the fight against counterfeit medicines and the entry into force of the Counterfeit Medicines Directive, which enables the verification of the authenticity of each individual medicine package [37,38]. In the case of the unit dispensing of antibiotics, verification would be possible from the manufacturer to the supplier, but not from the community pharmacy to the patient.

In addition, the former belief that it is extremely important to “complete the antibiotic therapy to the end”, which, for example, for CAP caused by atypical bacteria used to be 2 to 3 weeks, is now being abandoned. New knowledge about the advantages of a shorter antibiotic regimen leads to shortening of antimicrobial therapy, but it is still not included in the recommendations of all the relevant guidelines. Individualized antimicrobial therapy based on the specific needs of each individual patient could soon be expected [7,39,40]. 

Although all guidelines for the treatment of CAP state the approximate duration of therapy, they still emphasize that the duration of therapy should be determined according to the patient’s response to the prescribed therapy [7,41,42,43,44]. Prescribing and issuing fixed packages of antibiotics in this case inevitably lead to antibiotic residues, increased possibilities for their inappropriate use, and a consequent increase in antibiotic resistance, which emphasizes the importance of implementing the antimicrobial stewardship programs.

The unit dispensing of antibiotics and antimicrobial stewardship programs increases therapy costs. However, Smith et al. point out that there is no strategy that is too expensive when talking about antimicrobial resistance. Any cost and contribution to the containment of antimicrobial resistance is considered reasonable and justified because the battle will be the most expensive if lost [45].

Greater inclusion of all stakeholders is needed. For example, pharmacist-led antibiotic stewardship intervention can improve CAP guideline adherence both in hospitals and upon hospital discharge [46,47]. For example, clinical pharmacists audit of antibiotic order have shown to optimize antibiotic treatment duration and adherence to guidelines in CAP in an inpatient setting. Such intervention also supports the more frequent use of narrow-spectrum antimicrobials [48]. In another study, pharmacist-initiated modification to the duration of antibiotic treatment for CAP significantly reduced total antibiotic prescribing and shortened the treatment duration with the greatest impact on interventions on discharge prescribing. This intervention did not affect the patient readmission rates [49]. A study on 20,444 patients showed that, solely, a review of treatment prior to discharge was consistently associated with lower rates antibiotic overuse at discharge regardless of indication. Interestingly, the same study found that hospitals that had a preset antibiotic duration for CAP more frequently had antibiotic overuse at the discharge of patients with CAP [15].

Medication review of discharge prescribing does not have to be limited to the inpatient setting and may be performed by clinical pharmacists or community pharmacists during dispensing depending on the organization of the healthcare system. Pharmacist-developed intervention in the form of prescribing algorithms in an emergency department for the outpatient treatment of CAP resulted in improved guidelines adherence [50]. A number of stewardship measures in outpatient settings have been proposed, many of them including community pharmacists [51]. Implementing audits in feedback on antibiotic prescribing in primary care has shown to improve guideline adherence for the management of outpatient pediatric CAP [52].

Finally, we should be aware of additional factors leading to antibiotic waste. In addition to leftover antibiotics resulting from package sizes that do not match prescribed treatment durations, non-adherence to prescribed therapy by patients is another significant contributor. Non-adherence is a well-recognized public health challenge that requires comprehensive interventions in order to address it effectively. Enhancing patient education to improve understanding of the disease and therapy, along with employing behavioral techniques, can significantly improve medication adherence.

## 4. Materials and Methods

For the purpose of this study, criteria were developed to determine the accordance of approved antibiotic packages for treating CAP (criteria), which are based on recommendations from national guidelines for treating CAP. Additionally, a list of approved package sizes of antibiotics for oral use in the treatment of CAP in Croatia was compiled. Subsequently, the accordance of approved antibiotic packages with the number of antibiotic doses resulting from the specified criteria was determined.

Through a literature review, numerous international and national guidelines for treating CAP have been identified [7,41,42,43,44]. Considering that all guidelines emphasize the importance of local antimicrobial resistance when making decisions about pneumonia treatment, this study utilized the Croatian national guidelines [7].

From the guidelines, recommended active substances, pharmaceutical forms, strengths, dosing regimens, and durations of therapy have been established. Therapeutic options involving parenterally administered antibiotics were excluded, as this study focused on therapeutic possibilities for treating mild CAP.

If the guidelines recommended a range for the strength of the recommended antibiotic (for example, amoxicillin 500 mg to 1000 mg), for the purposes of this study, the minimum and maximum doses were considered.

Similarly, if the guidelines recommended a duration of therapy within a certain range (for example, from 7 to 10 days), for the purposes of this study, the shortest and longest duration of therapy were considered.

If the national guidelines did not specify the duration of therapy, the Summary of Product Characteristics (SmPC) on the official website of the Croatian Agency for Medicinal Products and Medical Devices (HALMED) was consulted for further information.

Furthermore, if a difference in antibiotic dosing was observed when consulting the SmPC compared to the national guidelines, both possibilities were considered for the purposes of this study.

Different oral pharmaceutical forms of the same active substance demonstrating identical pharmacokinetic properties were considered interchangeable. If the national guidelines did not specify the pharmaceutical form of the drug, and if different forms have different dosing regimens (for example, clarithromycin film-coated tablets and extended-release tablets), further details were consulted from the SmPC of the respective medications.

Furthermore, if the SmPC for the mentioned pharmaceutical forms indicated a different duration of therapy compared to the national guidelines, a broader range of duration of therapy was considered for the purposes of this study.

For the purposes of this study, it is assumed that the prescribed antibiotics led to improvement and successfully cured the infection, meaning that there was no need to discontinue the prescribed antibiotic and replace it with another within 72 h of initiation. Additionally, ideal patient adherence to the prescribed therapy is assumed.

Each therapeutic option was defined by the active substance, pharmaceutical form, strength, dosing interval, and duration of therapy.

In compiling the list of approved package sizes of antibiotics, all drugs from the ATC J01 group (Anatomical Therapeutic Chemical classification; preparations for the treatment of bacterial infections for systemic use) were primarily identified from the HALMED drug database [53] based on their names, which are also found in the national guidelines for the treatment of CAP. Then, all the available package sizes on the market in the Republic of Croatia for each drug were determined by reviewing the electronic product catalogs of the four largest wholesalers in Croatia (Medika, Medical Intertrade, Phoenix Farmacija, and Oktal Pharma).

Since this study focuses on the outpatient treatment of mild pneumonia, only oral pharmaceutical forms of antibiotics in all registered doses were included. Azithromycin 125 mg and 1000 mg and cefuroxime 125 mg were excluded due to the impracticality of their administration and low likelihood of prescribing those doses in practice.

Additionally, the list did not include pharmaceutical forms of antibiotics that do not have pre-specified dosage units by the manufacturer (such as powders for oral solutions), as well as drugs for which it was stated that they have not been placed on the market in Croatia or their supply has been permanently discontinued.

Different pharmaceutical forms demonstrating the same pharmacokinetic characteristics were considered interchangeable.

Medications with the same active ingredient, same strength, and same package size were considered to be one result regardless of the registered drug name.

The compiled list of approved package sizes of antibiotics for oral use in Croatia was compared with the established criteria.

**Figure 1:**
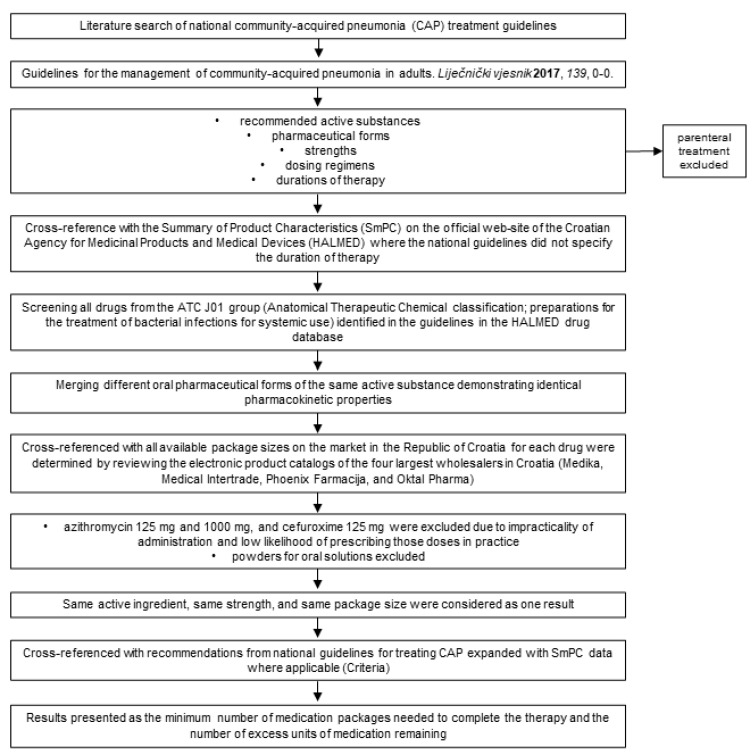
Study flowchart [7].

The results were presented as proposed therapeutic options and matched or mismatched packages. Matched packages were those that did not result in excess units of medication after comparison with the specified therapeutic options. Mismatched packages resulted in excess units of medication, and this excess, calculated based on the original number of units of medication in the package, was quantified and presented as a whole number. The results were presented as the minimum number of medication packages needed to complete the therapy and the number of excess units of medication remaining. A flowchart of the study is presented in Figure 1.

## 5. Conclusions

The results of this research showed a significant non-accordance of the approved antibiotic packages with the national guidelines for the treatment of CAP and, consequently, the creation of a large amount of residues of unit doses of antibiotics in the community. Given that antibiotic residues in the community greatly contribute to the development of antimicrobial resistance, which today is considered not only a health but also a social and economic problem, it is of great importance to implement effective solutions. It seems that the unit dispensing of antibiotic therapy could represent an effective solution in addition to antimicrobial stewardship programs in primary healthcare and that clinical pharmacists can significantly contribute to the prevention of the increase in antimicrobial resistance.

## Figures and Tables

**Table 1:** List of approved package sizes of antibiotics for oral use in Croatia, recommended for the treatment of community-acquired pneumonia (CAP).

Active Substance	Pharmaceutical Form	Strength	Number of Doses per Package
Azithromycin	Film-coated tablets, tablets for oral suspension	500 mg	3
Azithromycin	Film-coated tablets, hard capsules	250 mg	6
Amoxicillin	Film-coated tablets, tablets for oral suspension, hard capsules	500 mg	16
Amoxicillin	Film-coated tablets, tablets for oral suspension	1000 mg	16
Amoxicillin with clavulanic acid	Film-coated tablets, tablets for oral suspension/disintegrating oral tablets	875 mg + 125 mg	14
Amoxicillin with clavulanic acid	Film-coated tablets	875 mg + 125 mg	12
Cefuroxime axetil	Film-coated tablets	250 mg	10
Cefuroxime axetil	Film-coated tablets	500 mg	10
Cefuroxime axetil	Film-coated tablets	500 mg	14
Cefuroxime axetil	Film-coated tablets	500 mg	16
Doxycycline	Hard capsules	100 mg	25
Moxifloxacin	Film-coated tablets	400 mg	5
Moxifloxacin	Film-coated tablets	400 mg	7
Levofloxacin	Film-coated tablets	500 mg	10
Cefpodoxime	Film-coated tablets	100 mg	10
Cefpodoxime	Film-coated tablets	100 mg	20
Cefpodoxime	Film-coated tablets	200 mg	10
Cefpodoxime	Film-coated tablets	200 mg	20
Clarithromycin	Extended-release tablets	500 mg	7
Clarithromycin	Extended-release tablets	500 mg	14
Clarithromycin	Film-coated tablets	250 mg	14
Clarithromycin	Film-coated tablets	500 mg	14

**Table 2:** The accordance of antibiotic package sizes in Croatia with the criteria.

Active Substance	Package Size and Strength	Dosing Interval and Duration of Therapy	Number of Packages Needed to Complete the Therapy	Number of Excess Units	Accordance
Amoxicillin	16 × 500 mg	3 × 500 mg, 7 days	2	11	no
	16 × 500 mg	3 × 500 mg, 10 days	2	2	no
	16 × 500 mg	3 × 1000 mg, 7 days	3	6	no
	16 × 500 mg	3 × 1000 mg, 10 days	4	4	no
	16 × 1000 mg	3 × 1000 mg, 7 days	2	11	no
	16 × 1000 mg	3 × 1000 mg, 10 days	2	2	no
Cefuroxime axetil	10 × 250 mg	2 × 500 mg, 7 days	3	2	no
	10 × 500 mg	2 × 500 mg, 7 days	2	6	no
	14 × 500 mg	2 × 500 mg, 7 days	1	0	yes
	16 × 500 mg	2 × 500 mg, 7 days	1	2	no
Amoxicillin with clavulanic acid	12 × 1 g	2 × 1 g, 7 days	2	10	no
	12 × 1 g	2 × 1 g, 10 days	2	4	no
	14 × 1 g	2 × 1 g, 7 days	1	0	yes
	14 × 1 g	2 × 1 g, 10 days	2	8	no
Cefpodoxime	10 × 100 mg	2 × 200 mg, 7 days	3	2	no
	20 × 100 mg	2 × 200 mg, 7 days	2	12	no
	10 × 200 mg	2 × 200 mg, 7 days	2	6	no
	20 × 200 mg	2 × 200 mg, 7 days	1	6	no
Levofloxacin	10 × 500 mg	1 × 500 mg, 7 days	1	3	no
	10 × 500 mg	1 × 500 mg, 14 days	2	6	no
	10 × 500 mg	2 × 500 mg, 7 days	2	6	no
	10 × 500 mg	2 × 500 mg, 14 days	3	2	no
Moxifloxacin	5 × 400 mg	1 × 400 mg, 10 days	2	0	yes
	7 × 400 mg	1 × 400 mg, 10 days	2	4	no
Azithromycin	6 × 250 mg	1 × 500 mg, 3 days	1	0	yes
	3 × 500 mg	1 × 500 mg, 3 days	1	0	yes
Clarithromycin	14 × 250 mg, film-coated tablets	2 × 500 mg, 6 days	2	4	no
	14 × 250 mg, film-coated tablets	2 × 500 mg, 14 days	4	0	yes
	14 × 500 mg, film-coated tablets	2 × 500 mg, 6 days	1	2	no
	14 × 500 mg, film-coated tablets	2 × 500 mg, 14 days	2	0	yes
	7 × 500 mg, extended-release tablets	1 × 500 mg, 6 days	1	1	no
	7 × 500 mg, extended-release tablets	1 × 500 mg, 14 days	2	0	yes
	14 × 500 mg, extended-release tablets	1 × 500 mg, 6 days	1	8	no
	14 × 500 mg, extended-release tablets	1 × 500 mg, 14 days	1	0	yes
	7 × 500 mg, extended-release tablets	1 × 1000 mg, 6 days	2	2	no
	7 × 500 mg, extended-release tablets	1 × 1000 mg, 14 days	4	0	yes
	14 × 500 mg, extended-release tablets	1 × 1000 mg, 6 days	1	2	no
	14 × 500 mg, extended-release tablets	1 × 1000 mg, 14 days	2	0	yes
Doxycycline	25 × 100 mg	2 × 100 mg, 10 days	1	5	no

**Table 3:** Summary table of accordance of drug packs with the clinical guidelines.

Antibiotic	Matching *
Amoxicillin	0/6
Cefuroxime axetil	1/4
Amoxicillin with clavulanic acid	1/4
Cefpodoxime	0/4
Levofloxacin	0/4
Moxifloxacin	1/2
Azithromycin	2/2
Clarithromycin	6/12
Doxycycline	0/1

* matching calculated as matches/combinations.

## Data Availability

All data are presented in the manuscript.

## Supplementary Materials

The following supporting information can be downloaded at: https://www.mdpi.com/article/10.3390/antibiotics13060546/s1, Table S1: Criteria for determining the accordance of approved antibiotic packages and recommended antibiotic therapy for treating community acquired pneumonia (CAP).
