# Supplementary material for: Accordance of Registered Drug Packages with Guideline-Recommended Treatment Durations for Community-Acquired Pneumonia—A New Antibiotic Stewardship Target?
Source: Antibiotics (Basel). 2024 Jun 12;13(6):546. doi: 10.3390/antibiotics13060546 (PMC11201020; doi:10.3390/antibiotics13060546)
Supplement: Supplementary file 1 [file antibiotics-13-00546-s001.zip › antibiotics-3013839-supplementary.pdf]

**Supplementary file**

**Table S1.** Criteria for determining the accordance of approved antibiotic packages and recommended antibiotic therapy for treating community acquired pneumonia (CAP)

| Active Substance | Pharmaceutical Form | Strength | Dosing Interval | Duration of Therapy |
|---|---|---|---|---|
| Amoxicillin | film-coated tablets, tablets for oral suspension, hard capsules | 500 mg | 3 × 500 mg | 7 days |
| Amoxicillin | film-coated tablets, tablets for oral suspension, hard capsules | 500 mg | 3 × 500 mg | 10 days |
| Amoxicillin | film-coated tablets, tablets for oral suspension, hard capsules | 1000 mg | 3 × 1000 mg | 7 days |
| Amoxicillin | film-coated tablets, tablets for oral suspension, hard capsules | 1000 mg | 3 × 1000 mg | 10 days |
| Amoxicillin with clavulanic acid | film-coated tablets, tablets for oral suspension / disintegrating oral tablets | 1 g | 2 × 1 g | 7 days |
| Amoxicillin with clavulanic acid | film-coated tablets, tablets for oral suspension / disintegrating oral tablets | 1 g | 2 × 1 g | 10 days |
| Cefuroxime axetil | film-coated tablets | 500 mg | 2 × 500 mg | 7 days |
| Cefpodoxime | film-coated tablets | 200 mg | 2 × 200 mg | 7 days |
| Levofloxacin | film-coated tablets | 500 mg | 1 × 500 mg | 7 days |
| Levofloxacin | film-coated tablets | 500 mg | 1 × 500 mg | 14 days |
| Levofloxacin | film-coated tablets | 500 mg | 2 × 500 mg | 7 days |
| Levofloxacin | film-coated tablets | 500 mg | 2 × 500 mg | 14 days |
| Moxifloxacin | film-coated tablets | 400 mg | 1 × 400 mg | 10 days |
| Azithromycin | film-coated tablets, tablets for oral suspension | 500 mg | 1 × 500 mg | 3 days |
| Clarithromycin | film-coated tablets | 500 mg | 2 × 500 mg | 6 days |
| Clarithromycin | film-coated tablets | 500 mg | 2 × 500 mg | 14 days |
| Clarithromycin | Extended-release tablets | 500 mg | 1 × 500 mg | 6 days |
| Clarithromycin | Extended-release tablets | 500 mg | 1 × 500 mg | 14 days |
| Clarithromycin | Extended-release tablets | 500 mg | 1 × 1000 mg | 6 days |
| Clarithromycin | Extended-release tablets | 500 mg | 1 × 1000 mg | 14 days |
| Doxycycline | Hard capsules | 100 mg | 2 × 100 mg | 10 days |
